# W‐N‐C Tandem Catalytic Centers Mediating Efficient Charge Transfer for the Enhanced Radical‐Path U(VI) Photoreduction

**DOI:** 10.1002/advs.75637

**Published:** 2026-05-08

**Authors:** Zhiyao Wu, Jinhao Xu, Hao Fu, Yuxiang Deng, Nannan Wang, Wenhua Zhang, Peng Zhang, Zhiwei Huang, Zhenpeng Cui, Shu‐Xian Hu, Wangsuo Wu, Duoqiang Pan

**Affiliations:** ^1^ State Key Laboratory of Chemistry for NBC Hazards Protection Frontiers Science Center for Rare Isotopes School of Nuclear Science and Technology Lanzhou University Lanzhou China; ^2^ School of Mathematics and Physics University of Science and Technology Beijing Beijing China; ^3^ State Key Laboratory for Conservation and Utilization of Subtropical Agro‐bioresources College of Agriculture Guangxi University Nanning China; ^4^ State Key Laboratory of Featured Metal Materials and Life‐cycle Safety for Composite Structures MOE Key Laboratory of New Processing Technology for Nonferrous Metals and Materials Guangxi University Nanning China; ^5^ National Synchrotron Radiation Laboratory University of Science and Technology of China Hefei China

**Keywords:** lewis‐acidic site, radical‐path, U(VI) photoreduction, W‐N‐C catalytic center

## Abstract

Photocatalytic reduction of uranium is pivotal for environmental remediation and sustainable resource utilization. Nevertheless, the precise control over the reaction pathway and the concurrent optimization on charge separation efficiency remain formidable challenges. Herein, W─N─C coordination was fabricated through the incorporation of WO_3‐x_ with three‐dimensional ordered macropores g‐C_3_N_4_ (3DOM g‐C_3_N_4_). The tandem catalytic center simultaneously enhanced the intrinsic activity of g‐C_3_N_4_ and enabled an efficient radical reduction pathway of U(VI). The formation of W─N─C coordination and structural distortion of the tri‐s‐triazine units enhanced n → π^*^ and π → π^*^ transition, resulting in a narrowed bandgap and improved visible‐light harvesting. Moreover, the Lewis‐acidic W^6+^ sites promoted O_2_ adsorption, facilitating the selective generation of $O_{2}^{-}$. The radical‐mediated U(VI) reduction pathway was thermodynamically favored, where W sites acted as electron‐accepting centers, with the W─N─C coordination serving as a charge‐transfer channel under a built‐in electric field, enabling the Z‐scheme migration. Consequently, the optimized catalyst (CW‐2) exhibited a U(VI) removal rate of 98.6% (capacity of 557.56 mg/g) within 16 min, the removal efficiency was reduced to a minute scale, and the impractical oxygen exclusion was no longer a significant concern. This work established a paradigm for creating “all‐in‐one” photocatalytic active centers through targeted atomic‐scale interface design.

## Graphical abstract

This work constructed a tandem W−N−C center to simultaneously achieve the regulation of reaction pathways and optimization of charge separation efficiency. The Lewis‐acidic W^6+^ sites activated O_2_ to generate $O_{2}^{-}$, with enhanced charge separation via electronic modulation, steering U(VI) reduction down an efficient radical pathway. This synergy enabled minute‐scale uranium removal with the oxygen‐compatible character.

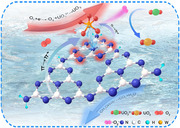

## 1 Introduction

The low greenhouse gas emissions and high energy density of nuclear energy make it a strong candidate for achieving carbon peak and carbon neutrality goals [1]. In the nuclear industry, uranium is utilized extensively. However, a considerable discrepancy exists between the available uranium reserves and the demand for this resource [2]. Conversely, the substantial volumes of uranium‐containing wastewater produced during nuclear processes constitute a grave threat to the ecological environment [3]. The utilization of solar‐driven photocatalytic reduction technology for the purpose of soluble U(VI) removal from water bodies has the potential to achieve two key objectives: the recovery of resources and the mitigation of water pollution [4].

The photocatalytic U(VI) removal efficiency is governed by the operative reaction pathway, thereby highlighting the critical importance of steering the reaction mechanism. The direct photoreduction of U(VI) is hampered by limited surface adsorption sites and the requisite anaerobic conditions, which is difficult to achieve and maintain in large‐scale or continuous‐flow wastewater treatment, thus hindering its further practical implementation [5, 6]. Although the indirect pathway of precipitating U(VI) via H_2_O_2_ (from oxygen reduction reaction, ORR) is oxygen‐tolerant [7], which requires catalyst nucleation sites on the surface. Consequently, accumulating uranium precipitates inevitably block the active sites, leading to rapid catalyst deactivation [8]. Furthermore, the generation of diverse byproducts (e.g.,·OH, ^1^O_2_ and $O_{2}^{-}$) during the ORR indicates low selectivity and leads to inefficient utilization of photogenerated electrons [9, 10]. Notably, U(VI) can be indirectly reduced by $O_{2}^{-}\;$ generated from the one‐electron oxygen reduction. This pathway not only utilizes photogenerated electrons more efficiently but also enables the reaction to proceed at the solid‐liquid interface, thereby preventing the occupation of active sites and facilitating their continuous regeneration.

Graphitic carbon nitride (g‐C_3_N_4_) as a metal‐free semiconductor, has garnered significant attention for photocatalytic U(VI) reduction due to its suitable bandgap (2.7 eV), low cost, feasible synthesis, sustainability, and high chemical durability [11, 12]. In particular, 3DOM g‐C_3_N_4_ exhibits structural advantages, including improved mass transport and abundant anchoring sites, making it a favorable candidate substrate for further modification. Similar structure‐induced benefits, such as increased specific surface area, have been demonstrated in hollow MOF architectures [13], underscoring the importance of tailored morphologies in heterogeneous catalysis. The photocatalytic activity of g‐C_3_N_4_ is determined by two intrinsic charge transport modes: the lone pair electrons of N atoms (n → π^*^) and the π bonding orbitals to antibonding π^*^ orbitals transition (π → π^*^) [14]. However, the highly localized *π*‐conjugated electrons, along with the symmetric tri‐s‐triazine structure, impede charge excitation of localized electrons and the long pair electrons of N atoms, constraining the overall photocatalytic efficiency [15]. Breaking this structural symmetry has recently been demonstrated as an effective strategy to modulate electronic states and enhance catalytic activity [16].

Therefore, simultaneously achieving high radical pathway selectivity and superior charge separation remains a formidable challenge. Many strategies have been implemented to promote the π → π^*^ and n → π^*^ transition, including but not limited to: heterojunction engineering [17], element doping [18], and morphology control [19]. Among these, M−N−C configurations offer a precise mean to tune catalytic interfaces through d−p orbital hybridization, which simultaneously modulates the electronic properties of the g‐C_3_N_4_ support and metal centers [20, 21]. Critically, the selection of the metal center dictates the adsorption mode of target molecules. For instance, creating strong Lewis‐acidic sites favors the single‐line Pauling‐type adsorption of O_2_ [22], effectively steering the ORR pathway toward the selective generation of $O_{2}^{-}$ [23], which is beneficial for the efficient radical path U(VI) reduction. W(VI) with the 5d^0^ electronic configuration [24] can accept lone pair electrons from O 2p orbitals to promote the free‑radical pathway and exhibits advantages such as environmental friendliness and cost‐effectiveness. Consequently, it appears well‐suited for constructing W─N─C coordination to simultaneously achieve regulation of the g‐C_3_N_4_ *π*‐conjugated structure and the formation of Lewis‐acidic sites.

Inspired by the spatial separation strategy employed in tandem electrocatalysts [25], herein, W−N−C tandem catalytic centers were constructed by integrating WO_3‐x_ onto 3DOM g‐C_3_N_4_. XAFS and sXAS analyses confirmed the formation of W─N coordination and the distortion of the symmetric tri‐s‐triazine structure. The higher electronegativity of nitrogen and the differences in Fermi levels drove the electron transfer from W to N, which promoted electron delocalization of *π*‐conjunction within the g‐C_3_N_4_ framework and injected electrons into the N atoms, thereby enhancing both n → π^*^ and π → π^*^ transitions. Furthermore, the Lewis‐acidic W^6+^ 5d^0^ sites facilitated Pauling‐type O_2_ adsorption via W/O 5d/2p orbital hybridization, as supported by DFT calculations, effectively promoting $O_{2}^{-}\;$ mediated U(VI) reduction pathway. Differential charge analysis suggested that the g‐C_3_N_4_ served as an electron supplier, while W sites acted as catalytic centers, establishing a tandem catalytic system with a clear division of functions. An intrinsic electric field induced by charge redistribution—analogous to that reported in hierarchical sulfide/phosphide heterostructures [26]—promoted the excited charge transfer from g‐C_3_N_4_ to WO_3‐x_ under illumination, following a Z‐scheme migration mechanism. As a result, the optimized CW‐2 sample achieved a high removal rate under U(VI) concentration of 4 × 10^−4^ m within 16 min, which were 6.85 and 5 times than those of g‐C_3_N_4_ and WO_3‐x_, respectively, demonstrating strong anti‐interference capability in complex water matrices. This work proposed a “all‐in‐one” strategy to simultaneously enhance the photocatalytic activity of g‐C_3_N_4_ and realize an efficient radical‐mediated U(VI) reduction pathway.

## 2 Results and Discussion

### 2.1 Synthesis and Characterization

**FIGURE 1:**
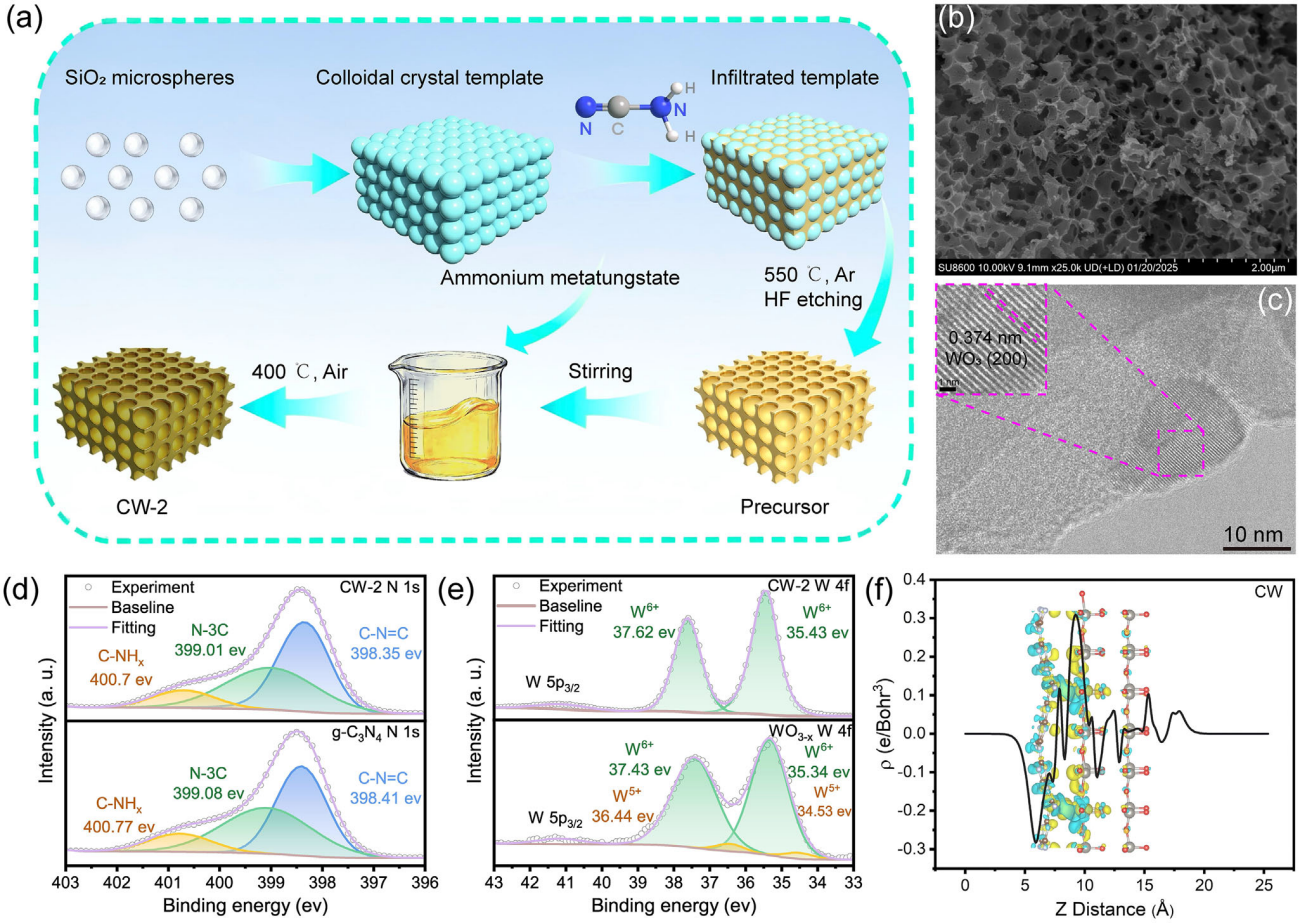
(a) Schematic diagram of the synthesis process of CW‐2. (b) SEM and (c) HRTEM images of CW‐2. High‐resolution XPS spectra of (d) N 1s and (e) W 4f. (f) Charge‐density difference of CW (yellow area: electron accumulation, blue area: electron depletion).

As shown in Figure 1a, the three‐dimensionally ordered g‐C_3_N_4_ (3DOM g‐C_3_N_4_) was synthesized through a template method. Briefly, cyanamide solution was thoroughly immersed into the ordered SiO_2_ array, following which the complexes were calcined at 550 °C. Thereafter, the 3DOM g‐C_3_N_4_ was obtained by removing the SiO_2_ using 5% HF solution and calcined at 400 °C. The 3DOM g‐C_3_N_4_@WO_3‐x_‐*x* (CW‐*x*) was synthesized through slow oxidation of W adsorbed on g‐C_3_N_4_. The unsaturated coordination state of oxygen resulted in the electrons enrichment on W sites, leading to electron transfer from W to g‐C_3_N_4_ through W−N hybridization. This was attributed to the greater negative electronegativity of N compared to that of O, consequently, the charge transfer W─N─C coordination was established. X‐ray diffraction (XRD) patterns confirmed the successful preparation of hybrid samples. Specifically, the presence of peaks at approximately 10° and 27° was indicative of the (100) Bragg peak and π‐ − π interlayer stacking (200) facet of g‐C_3_N_4_, respectively [27]. WO_3‐x_ was found to exist predominantly in a monoclinic crystal structure (PDF#72‐0677) [28], while the characteristic peaks at about 14° and 36° attributed to (100) and (201) facets of the hexagonal phase was also observed [29]. After integration, no obvious characteristic peak of WO_3‐x_ was observed for the XRD patterns of CW‐*x*, which might be attributed to the low loading capacity (Figure S1). Surface chemical groups of samples were identified by Fourier transform infrared spectroscopy (FT‐IR). The broad peak at 3400 cm^−1^ was induced by the ─OH stretching vibrations of surface adsorbed water molecule [30], the peak at 2168 cm^−1^ was deemed as the stretching vibration of ─C≡N, and the peak at 810 cm^−1^ was attributed to the bending vibrations of the heptazine ring. Furthermore, a range of peaks between 1200 and 1650 cm^−1^ were identified, which were attributed to the stretching vibrations of the C─N heterocycle (Figure S2) [31]. The specific surface area was determined through N_2_ adsorption‐desorption isotherms; the BET surface area of g‐C_3_N_4_ and CW‐2 was 38.661 and 37.275 m^2^/g, respectively. It was evident that no discernible variation had been observed prior to and following the integration of WO_3‐x_, thereby suggesting that physical adsorption did not constitute a primary factor influencing the efficiency of U(VI) removal. Pore distribution data suggested that both g‐C_3_N_4_ and CW‐2 possessed macroporous characteristics (Figure S3).

Scanning electron microscopy (SEM) validated that the ordered microporous morphology of g‐C_3_N_4_ was successfully fabricated through SiO_2_ template (Figure S4), and no discernible discrepancy was evident in the morphology of CW‐2 after WO_3‐x_ integration (Figure 1b). The transmission electron microscopy (TEM) image revealed the ultrathin nanosheet structure of CW‐2 (Figure S5). Elemental mapping demonstrated the uniform distribution of C, N, W, and O in CW‐2, indicating the successful integration (Figure S7). WO_3‐x_ had been found to exhibit a nanoparticulate morphology with dimensions of approximately 50 nm. In addition, lattice fringes of 0.374 nm were observed in a high‐resolution TEM image, which was indicative of the (020) facets of WO_3_ (Figure S6). As demonstrated in Figure 1c, the presence of WO_3‐x_ nanoparticles in CW‐2 was identified by the distinct lattice fringes of 0.374 nm as evidenced by the high‐resolution TEM image [32]. However, due to the poor crystallinity, no discernible lattice stripes characteristic of g‐C_3_N_4_ were observed.

X‐ray photoelectron spectroscopic (XPS) analysis was employed to investigate the chemical state and charge transfer during the formation of W─N─C coordination. All elements of g‐C_3_N_4_ and WO_3‐x_ were observed of CW‐2 in XPS survey spectrum, suggesting the successful integration (Figure S8a). C 1s orbital was divided into three peaks, which are C─C/C═C, C─NH_x,_ and N─C═N chemical bonds at the bonding energy of 284.8, 286.18, and 288.36 eV, respectively for g‐C_3_N_4_ (Figure S8b) [33]. These peaks were 284.8, 286.14, and 288.3 eV for C 1s XPS spectrum of CW‐2, the introduction of WO_3‐x_ resulted in a shift toward the lower binding energy of the C 1s orbital. The N 1s high‐resolution XPS spectrum of g‐C_3_N_4_ was deconvoluted into three peaks, with the binding energy of 398.41, 399.08, and 400.77 eV are attributed to the sp^2^ hybridization N in C─N═C, sp^3^ bridging N in N−3C and ─NH&NH_2_ bonding, respectively [34]. These three peaks of CW‐2 were 398.35, 399.01, and 400.1 eV respectively, a shift toward lower binding energy was observed, which was analogous to the shift observed for the C 1s orbital (Figure 1d) [33]. The presence of mixed valence was confirmed for the W element in WO_3‐x_, as evidenced by the W 4f spectrum. Two peaks at the binding energy of 35.34 and 37.43 eV were attributed to W 4f_2/7_ and W 4f_2/5_ of W^6+^, while peaks at 34.53 and 36.44 eV corresponded to W 4f_2/7_ and W 4f_2/5_ of W^5+^ (Figure 1e) [32]. The incomplete oxidation process resulted in unsaturated oxygen coordination with surface reduced state sites of WO_3‐x_. The more electronegative element N, however, tended to compete for the electrons with O atom when bonded to W, resulting in the enhancement of the covalency between N 2p and W 5d orbitals. Following the integration process, the characteristic peak of W^5+^ was no longer evident. The enhancement of the binding force on electrons was observed to manifest as a higher binding energy in the W4f XPS result. Incomplete oxidation and electron competition at the N‐sites had been demonstrated to enhance the Lewis‐acidity of W sites. This, in turn, enabled precise recognition and adsorption of O_2_ during the catalysis process through capturing lone pair electrons from O 2p. A significant disparity was observed in the oxygen species present within the as‐prepared samples. The presence of two distinct peaks, indicative of a WO_3‐x_ lattice and surface‐adsorbed oxygen species, was detected at binding energies of 530.31 and 531.06 eV, respectively (Figure S8c) [35]. Two peaks attributed to C─O bonding, surface adsorbed ─OH were observed for g‐C_3_N_4_, located at the binding energy of 533.45 and 531.16 eV, respectively. Additionally, CW‐2 exhibited all characteristic peaks of both g‐C_3_N_4_ and WO_3‐x_, which were C─O bonding of g‐C_3_N_4_ framework (533.1 eV), surface adsorbed ─OH (531.07 eV), and lattice oxygen of WO_3‐x_ (530.27 eV), indicating the successful incorporation [36, 37].

To investigate the metal‐support interaction, charge density difference was obtained through DFT calculation, the optimized structural model of g‐C_3_N_4_, WO_3‐x,_ and CW‐2 were established (Figure S9a–c). The overlap of the dispersion of charge between the conduction band minimum (CBM) and the valence band maximum (VBM) of g‐C_3_N_4_ and WO_3‐x_ resulted in a stable state, which was challenging to excite (Figure S10). As demonstrated in Figure 1f, the introduction of WO_3‐x_ was shown to induce electron delocalization of g‐C_3_N_4_, resulting in charge depletion on the W atom and accumulation on the N atom within the contact area. The work function of WO_3‐x_ was calculated to be 3.311 eV, which is 4.903 eV for g‐C_3_N_4_. This discrepancy in work function indicated that WO_3‐x_ possess a higher Fermi level (Figure S11). Consequently, when a heterojunction was formed, charge would migrate from WO_3‐x_ to g‐C_3_N_4_ to promote the equilibrium of Fermi levels. The distortion observed in the electronic arrangement was indicative of an inherent electronic field between the interface of WO_3‐x_ and g‐C_3_N_4,_ attributing to charge balancing induced by differences in electronegativity and Fermi level. The built‐in electron field reduced the energy barrier for charge separation, and the charge‐depletion Lewis‐acidic W^6+^ sites accepted lone pair electrons from 2p orbital of O_2_ to enhance the adsorption through 5d/2p hybridization. Of particular significance was the potential for the disrupted localized *π*‐electrons to exhibit heightened activity in terms of charge migration.

### 2.2 Photocatalytic U(VI) Removal Performance

**FIGURE 2:**
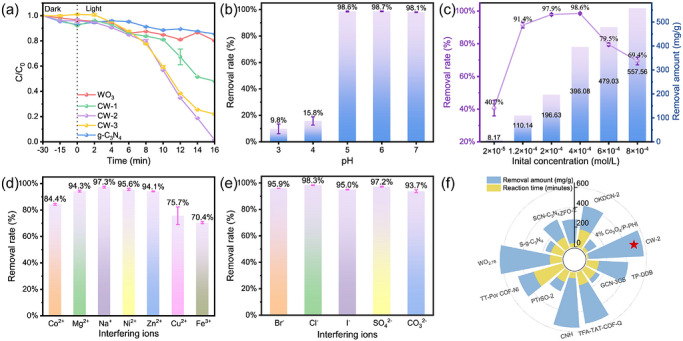
(a) Photocatalytic U(VI) removal performance of prepared samples (Conditions: C_U(VI)_ = 4 × 10^−4^ mol/L, C_MeOH_ = 8 VOL%, m/V = 0.5 g/L, T = 293 K, pH = 5). Photocatalytic U(VI) removal performance of CW‐2 under different (b) pH values, (c) initial U(VI) concentration, (d) interfering cations (C_C_: C_U_ = 1:2), and (e) interfering anions (C_A_: C_U_ = 1:2). (f) Comparison of photocatalytic U(VI) removal efficiency for CW‐2 with other photocatalysts.

The photocatalytic U(VI) removal efficiency of the as prepared samples was investigated at pH = 5, using MeOH as the h^+^ sacrificial agent and with the presence of oxygen. Prior to the irradiation process, the suspension was stirred in the dark at first in order to achieve adsorption equilibrium. The concentration of U(VI) exhibited minimal change at the dark reaction stage, indicating that physical adsorption was not the primary mechanism responsible for U(VI) removal. Volcanic trends were observed for the removal efficiency of U(VI), the integration of WO_3‐x_ resulted in the enhancement of photocatalytic activity, which reached a peak for CW‐2. However, further addition of WO_3‐x_ for CW‐3 led to a decline in activity. It can be hypothesized that excessive WO_3‐x_ may cover the active sites of g‐C_3_N_4_, thereby forming carrier complex centra and resulting in a decrease in efficiency. The highest U(VI) removal efficiency of 98.6% and the turnover frequency (TOF) of 0.08 h^−1^ was observed for CW‐2 within 16 min of irradiation, which was 14.4%, 19.7%, 51.9% and 78% for g‐C_3_N_4_, WO_3‐x_, CW‐1, and CW‐3, respectively (Figure 2a). It was noteworthy that during the initial 6 min of light irradiation, the U(VI) concentration demonstrated a more gradual change, while a substantial decrease occurred at the ninth minute. This phenomenon was attributable to the fact that the oxygen reduction reaction predominantly occurred in the initial stage, at which point photogenerated electrons reduced the dissolved oxygen to $O_{2}^{-}$ in water, while the U(VI) was further reduced by $O_{2}^{-}$.

Moreover, pH showed considerable influence on the surface charge of the catalyst and the prevailing species of U(VI) in water bodies [38]. As demonstrated in Figure S12, the surface charge of CW‐2 became increasingly negative as the pH value was altered from 2 to 7, attaining a zero charge point at a pH of about 4.6. At this point, the repulsive force between CW‐2 and ${\mathrm{UO}}_{2}^{2+}$ was diminished. The impact of pH was investigated by changing the pH from 3 to 7 using CW‐2 as a catalyst. As shown in Figure 2b, the photocatalytic activity was found to be minimal at pH 3 and 4. This phenomenon can be attributed to two primary factors, firstly, the abundance of H^+^ ions present competed with ${\mathrm{UO}}_{2}^{2+}$ and O_2_ for photogenerated electrons; secondly, there was charge repulsion between the positively charged surface catalyst and the positively charged ${\mathrm{UO}}_{2}^{2+}$ at these pH values [30]. In the range of 5–7 on the pH scale, the surface of CW‐2 became increasingly more negatively charged. This, in turn, led to an increase in the charge attraction to ${\mathrm{UO}}_{2}^{2+}$ and a concomitant tendency toward higher removal rates.

In natural waters, the existence of competing anions and cations is inevitable, and these in turn interfere with the efficiency of photocatalytic reduction of U(VI). Therefore, the effects of different anions and cations on the removal efficiency of U were investigated. CW‐2 exhibited adequate resistance to interference, with the exception of the presence of Co^2+^, Fe^3+,^ and Cu^2+^, where the uranium removal efficiencies were diminished to 84.4%, 75.7%, and 70.4%, respectively, for an equivalent reaction time, amongst the numerous interfering metal cations (Figure 2d). This phenomenon has been attributed primarily to the presence of the competition of adsorption sites between anions, O_2,_ and U(VI), in conjunction with the competition toward photogenerated electrons. However, the addition of interfering anions showed only a negligible effect on uranium removal efficiency, in which the addition of ${\mathrm{CO}}_{3}^{2-}$ caused a slight reduction in the removal of uranium (Figure 2e). This phenomenon may be attributable to the incorporation of ${\mathrm{CO}}_{3}^{2-}$, which impacted the configuration of U(VI) within the solution, resulting in the transformation of ${\mathrm{UO}}_{2}^{2+}$ into uranyl‐carbonate complexes. The latter possessed a more negative reduction potential, thereby augmenting the resistance to the reductive extraction [39].

As shown in Figure 2c The removal efficiency of CW‐2 was evaluated for the solutions with different initial U(VI) concentrations. In the instance of a low concentration of 2 × 10^−5^ mol/L, CW‐2 exhibited a low removal efficiency of U(VI), with a value of only 40.7%. However, as the U(VI) concentration increasing, the removal rate increased concomitantly, reaching a maximum at a concentration of 4 × 10^−4^ and then beginning to decline. In low concentrations, the U(VI) was challenging to be identified and reduced by active radicals or adsorbed by active sites. Furthermore, at elevated concentrations, the active radicals generated from ORR were inadequate in terms of their capacity to react with U(VI) in a timely manner, and the active sites would be occupied by uranium species. As illustrated in Figure 2f and outlined in Table S1, a comparative analysis was conducted to assess the photocatalytic U(VI) removal capacity of CW‐2 and several representative catalysts. As a result, CW‐2 displayed competitive photocatalytic U(VI) removal efficiency, both in terms of reaction time and removal rate.

The experimental investigation of the economy and stability of the catalyst was conducted using cycling experiments. Subsequent to each photocatalytic experiment, the catalyst was immersed in a 0.1 m Na_2_CO_3_ solution and stirred for a period of 12 h with the objective of removing uranium species that had been adsorbed on the surface. Following this, the material was dried and utilized in the subsequent cycling experiment. It was demonstrated that the catalytic activity was maintained at a consistent level of 92.1% even after four cycles, thus exhibiting excellent stability (Figure S13).

### 2.3 Local Environment and Structure‐Activity Relationship Analyses

**FIGURE 3:**
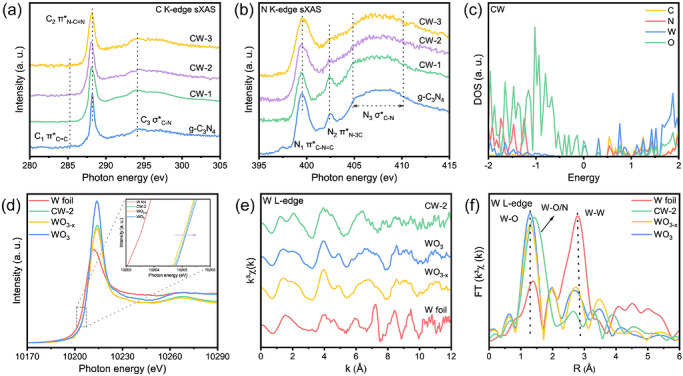
Synchrotron‐radiation sXAS spectra of (a) C K‐edge and (b) N K‐edge. (C) DFT calculated pDOS of CW‐2. (d) W L_3_‐edge XANES spectrum. (e) EXAFS k‐space function of W L_3_‐edge. (f) k^3^‐weighted FT‐EXAFS spectrum of W L_3_‐edge.

The utilization of soft X‐ray absorption spectroscopy (sXAS) as a surface‐sensitive method was undertaken to facilitate a more profound comprehension of the C, N hybridization. Both C and N K‐edge spectra were indicative of the electron transfer from the core 1s orbital to the unoccupied 2p orbital. As illustrated in Figure 3a, the region of C 1s to π^*^ and σ^*^ transitions were observed. Peak at about 285.3 eV derived from the out‐of‐plane C═C 1s to 2p2 antibonding transition was indicative of the presence of a graphitic feature [40]. The highest peak, observed at 288.3 eV, was ascribed to the 1s to 2sp^2^ π^*^ transition of N─C═N hybridization [41], which corresponded to the triazine ring. The long region located from approximately 293 to 300 eV was the in‐plane 1s to 2sp^3^ σ^*^ transition of C─N bonding orbital [42]. The introduction of WO_3‐x_ resulted in a shift in energy toward a lower direction and a decrease in the intensity of the C_1_ peak. This observation suggested the occurrence of charge accumulation on g‐C_3_N_4_ and a concomitant decrease in the number of unoccupied 2p states, which enhanced the covalency between C and N. In consideration of the N K‐edge spectra, the peaks observed at 400 and 402 eV, in conjunction with the extensive region extending from 405 to 412 eV, served as indicative markers for the transition from N 1s to the 2sp^2^ π^*^ orbital of C═N─C hybridization, sp^3^ hybrid tertiary N bonding to carbon atoms (N−3C), and the 2p σ^*^ C─N configuration (Figure 3b) [41, 43]. The N_1_ peak exhibited 0.1 eV shift toward lower energy, and intensity decreased after integration with WO_3‐x_, indicating lower energy was needed for the excitation of core electrons to 2p state and the more occupied 2p orbital. As indicated by the W 4f XPS and sXAS results, a redistribution of charge occurred between WO_3‐x_ and g‐C_3_N_4_, both the C and N K‐edges demonstrated a propensity for charge accumulation. It was important to note that the charge transfer‐induced W/N 5d/2p hybridization had a negative effect on the covalency degree of the high‐energy antibonding out‐of‐plane N−3C sp^3^ orbital corresponding to tri‐s‐triazine units, resulting in the enhancement of n → π^*^ transitions. The enhanced orbital hybridization between W and N promoted electron delocalization, constructing an interface built‐in electric field, and the asymmetric charge centra played a role in facilitating charge migration after excitation.

X‐ray absorption fine structure (XAFS) was utilized to obtain information regarding the chemical state and local coordination information of the 5d W atom. As demonstrated in Figure 3d, the white line (WL) peak at approximately 10210 eV of the W L_3_‐edge X‐ray absorption near edge structure (XANES) spectrum was indicative of the 2p_3/2_ to vacant 5d orbital transition of W [44]. The WL peak intensity exhibited a decrease in accordance with the order of WO_3_, CW‐2, WO_3‐x,_ and W foil. This phenomenon can be interpreted as an indication of a gradual decrease in the unoccupied 5d orbital. Moreover, the absorption edge of WO_3‐x_ and CW‐2 was located between the W foil and WO_3_, in close proximity to WO_3_. This finding suggested that transmission‐mode XAFS, which probed bulk‐sensitive information, revealed that W in WO_3‐x_ and CW‐2 existed in mixed +5 and +6 oxidation states. The greater electronegativity of nitrogen and the higher Fermi level of WO_3‐x_ resulted in the electron drainage of the W atom. Consequently, the WL intensity increased, and the absorption edge of CW‐2 shifted toward higher energy relative to that of pristine WO_3‐x_.

A high signal‐to‐noise ratio and a similar waveform was observed for CW‐2 and tungsten oxides in K space function, indicating their similar chemical environment (Figure 3e) [45]. The Fourier transformed *k^3^*‐weighted W L_3_‐edge extended X‐ray absorption fine structure (EXAFS) spectrum of the W foil exhibited a sharp peak at 2.9 A, representing the W─W bond, a weak peak at 1.4 in the first shell was attributed to the W─O bond, indicating the slight oxidation of the surface (Figure 3f). Focusing on the first shell layer, the W─O path of WO_3_ and WO_3‐x_ located at 1.28 A, while the lower peak intensity of WO_3‐x_ suggested the unsaturated coordination environment between W and O. Interestingly, the coordinate bond formed between W and N led to the enhancement of coordination strength in the first shell of CW‐2, and a shifting of the peak toward higher length caused the slightly longer W─N bond length than that of W─O bond [46, 47], thus the formation of W─N─C configuration was confirmed. The W─N─C coordination effectively restructured the electronic landscape of g‐C_3_N_4_. It not only constructed an efficient charge‐transfer channel but also enabled modulation of *π*‐conjugated frameworks. This delocalization introduced intermediate energy states that alleviate quantum confinement, thereby enhancing the π → π^*^ transition and overcoming the constraint of in‐plane charge migration [34].

As demonstrated by the projected density of states results, the CBM of g‐C_3_N_4_ was predominantly occupied by the C 2p orbital, while the VBM consisted of the N 2p orbital. The observation of a broad forbidden band indicated the wide bandgap and the semiconductor nature of g‐C_3_N_4_. In the case of WO_3‐x_, the O 2p orbital contributed to the VBM, while the W 5d orbital contributed to the CBM. The DOS waves were shown to cross the Fermi level and were not located in the zero DOS region. This was indicative of the metal properties of the semiconductor (Figure S14a,b) [48]. A prominent d‐p hybridization between W 5d and N 2p was observed, and the CBM consists of W 5d orbital, indicating the potential electron acceptor of W sites after photoexcitation for the case of CW‐2 [49]. Furthermore, a downshift of N 2p orbital in the CBM region and impurity states appeared at the forbidden band was observed. The intrinsic light‐harvesting capability was significantly enhanced through the construction of W─N─C coordination, which promoted the π → π^*^ transition, leading to a narrowed bandgap and more efficient excitation of VB electrons to the CB (Figure 3c). In summary, the constructed W─N coordination effectively disrupted the symmetric geometry of the tri‐s‐triazine units and enriched electron density on nitrogen atoms, thereby promoting the n → π^*^ transition. Concurrently, strong 5d/2p orbital hybridization extended the *π*‐conjugated frameworks, facilitating the π → π^*^ transition. Such electronic restructuring collectively enhanced the excitation of charge carriers across the bandgap, particularly activating low‐energy optical transitions, which led to markedly improved light utilization and charge separation efficiency.

In pristine g‐C_3_N_4_, the localized *π*‐electron cloud results in a uniform charge distribution, leading to weak and non‐selective O_2_ affinity at various N and C sites. In stark contrast, the introduction of W─N─C coordination orchestrated a pronounced charge redistribution. This created strong Lewis‐acidic W^6+^ sites, which are characterized by empty 5d orbitals. These sites acted as electron acceptors for the lone pair electrons of O_2_, thereby strengthening the adsorbate‐catalyst interaction. As shown in the O_2_ adsorption model (Figure S15a–c), O_2_ preferentially adsorbed onto these W sites in the CW‐2 composite rather than the g‐C_3_N_4_ matrix. This process established a Pauling‐type adsorption configuration, which directed the ORR pathway through a selective 2e^−^ transfer, ultimately favoring $O_{2}^{-}$. production [23]. On the other hand, the robust W−N−C orbital hybridization established a charge transfer channel that facilitates charge migration from g‐C_3_N_4_ to WO_3‐x_ under irradiation, thereby enhancing carrier utilization efficiency.

### 2.4 Photoelectrochemical Properties

**FIGURE 4:**
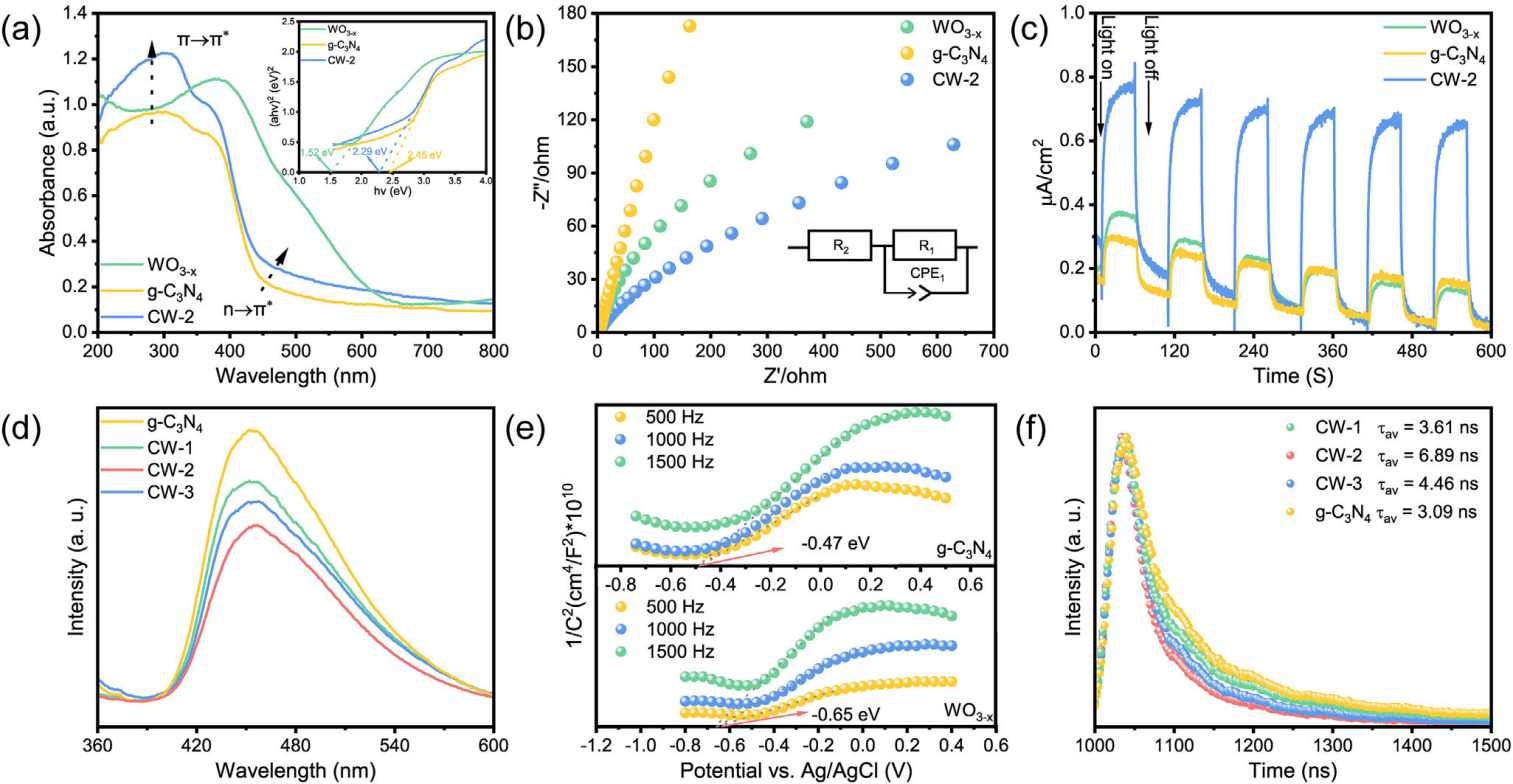
(a) UV–vis DRS and the calculated bandgaps (insert). (b) EIS plots. (c) Transient photocurrent density. (d) PL spectrum. (e) Mott–Schottky curves. (f) TRPL spectrum.

As shown in Figure 4a, g‐C_3_N_4_ exhibited relatively weak light utilization rate, with the absorption band occurring mainly in the UV range. The adsorption band from 200 to about 420 nm represented the C─N═C sp^2^ hybridization induced π → π^*^ transition of C─N coordination in the heptazine ring. The adsorption band in the visible light range was weak for g‐C_3_N_4_ from 420 to 650 nm, which was attributed to the lone pair electrons n → π^*^ transition [31]. WO_3‐x_ exhibited good absorption intensity within the UV and visible light regions, accompanied by a shoulder peak observed at approximately 420 nm. After integration, there was an enhancement in both visible and UV light utilization rate of CW‐2 in comparison with that of pristine g‐C_3_N_4_. The formation of W─N─C coordination induced the variations of the local coordination of g‐C_3_N_4_ through W─N 5d/2p hybridization and then promoted the π → π^*^ transition. Furthermore, the fracturing of the optimal symmetrical of tri‐s‐triazine‐based configuration [15], as demonstrated by the decreased N−C3 intensity in N K‐edge sXAS results, expedited the n → π^*^ transition of lone pair electrons in N. Tauc plots inset in Figure 4a demonstrated the bandgap energy (E_g_) of catalysts, the E_g_ of g‐C_3_N_4_, CW‐2 and WO_3‐x_ was calculated to be 2.45, 2.29 and 1.52 eV, respectively. The introduction of WO_3‐x_ improved the utilization rate of light, optimized the band structure, and thus reduced the energy barrier for electron transitions. The smallest radius of CW‐2 compared to that of g‐C_3_N_4_ and WO_3‐x_ observed in electrochemical impedance (EIS) spectra was indicative of reduced charge transfer resistance (Figure 4b). The separation capability of photogenerated carriers was evaluated using transient photocurrent response and photoluminescence spectroscopy (PL). As expected, CW‐2 exhibited the highest photocurrent intensity (Figure 4c) and the lowest PL intensity (Figure 4d). The W─N─C coordination constructed the charge transfer channel, and the internal electronic field increased the energy barrier for the composition of photoinduced carriers. As the direct evidence, the lifetimes of charge carriers of samples were estimated through time‐resolved photoluminescence spectroscopy (TR‐PL) measurements, the average fluorescence lifetimes (τa) of g‐C_3_N_4_, CW‐1, CW‐2 and CW‐3 were 3.09, 3.61, 6.89 and 4.46 ns, respectively (Figure 4f). The constructed of asymmetric charge center reduced the recombination of photo‐generated electrons and holes, allowing more charge to directly act on the target reactants and minimizing energy loss.

The band structure was calculated using Tauc plots and Mott–Schottky curves. The Mott–Schottky tests were performed on each sample thrice at frequencies of 500, 1000, and 1500 Hz (Figure 4e). The positive slope of the test results indicated that both g‐C_3_N_4_ and WO_3_ were n‐type semiconductors. The flat band potential of g‐C_3_N_4_ and WO_3‐x_ were −0.47 and −0.65 eV (vs. Ag/AgCl), which were −0.27 and −0.45 eV (vs. NHE). Cause the conduction band potential (E_CB_) of n‐type semiconductor was 0.1 eV greater than that of the flat band [50], therefore the E_CB_ of g‐C_3_N_4_ and WO_3‐x_ were −0.37 and −0.55 eV (vs. NHE). According to the formulation of E_g_ = E_VB_ − E_CB_ [51], the E_VB_ of g‐C_3_N_4_ and WO_3‐x_ were calculated to be 2.08 and 0.97 eV (vs. NHE) respectively. The reduction potential was high enough to reduce O_2_ to $O_{2}^{-}$ (−0.33 eV vs. NHE) and ${\mathrm{UO}}_{2}^{2+}$ to UO_2_ (0.41 eV vs. NHE) [52].

### 2.5 Dynamic Charge Transfer and Catalytic Mechanism Analyses

The charge transfer mode was investigated through XPS of CW‐2 after stability test, and the corresponding spectra were shown in Figure S16. It was demonstrated that significant changes in binding energy were observed for W and N elements, thus indicating that the redox reaction was primarily mediated by W and N. In particular, after the photocatalytic reaction, the W atom demonstrated an increase in electron density, while the reverse phenomenon was observed for the N atom in CW‐2. The electronegativity difference led to a tilting of the electron cloud toward g‐C_3_N_4_ after integration, thereby establishing an internal electric field at the heterojunction interface. In the presence of an internal electric field, the migration of electrons from the CB in g‐C_3_N_4_ toward WO_3‐x_ was observed under light excitation. This process resulted in charge enrichment at the W sites and subsequent acting on the reduction of oxygen. It was evident that the charge transfer mode conformed to the Z‐type mode in accordance with the staggered band structure of g‐C_3_N_4_ and WO_3‐x_ (Figure S18). This configuration fostered charge separation and amplified the redox potential in comparison to the performance of individual materials.

**FIGURE 5:**
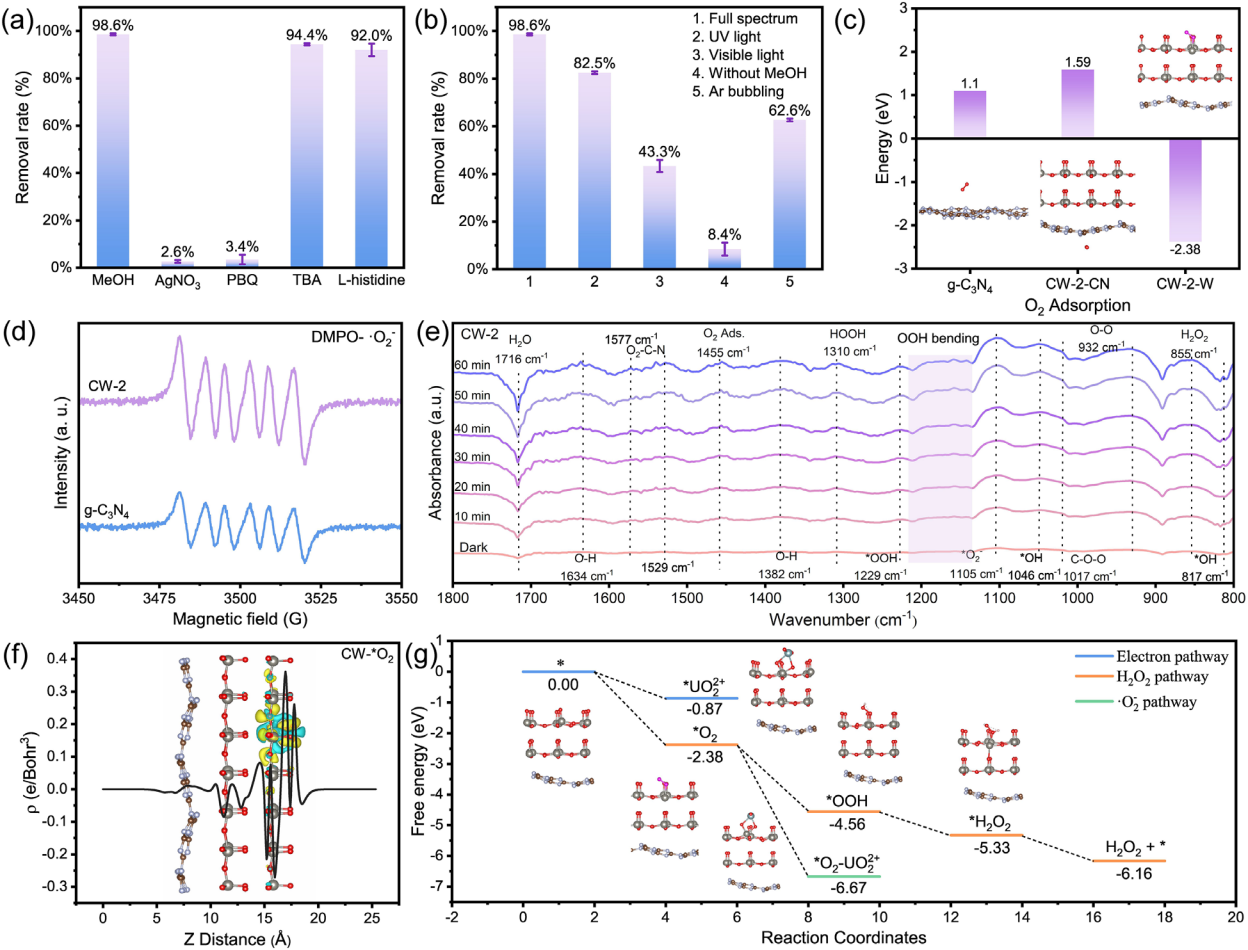
(a) Photocatalytic U(VI) removal performance of CW‐2 under (a) different scavengers, (b) different reaction conditions within 16 min. (c) Optimized O_2_ adsorption model on g‐C_3_N_4_ and CW‐2 and the corresponding adsorption energy. (d) EPR signal of DMPO O_2_^−^ of g‐C_3_N_4_ and CW‐2. (e) In situ DRIFTS spectrum of CW‐2 for the ORR reaction. (f) Charge density differences of the O_2_ adsorbed on CW‐2. (g) Gibbs free energy diagrams of complexing and reduction reaction between CW‐2 and uranyl obtained through DFT calculation.

In the presence of oxygen, reactive oxygen species (ROS) generated during the ORR often contribute to uranium extraction. Thus, chemical quenching experiments were performed upon CW‐2, MeOH, AgNO_3_, benzoquinone (p‐BQ), tert‐butanol (TBA), and L‐histidine were used to capture h^+^, e^−^, $O_{2}^{-}$,·OH and ^1^O_2_, respectively. Notably, the removal rate reduced to 2.6% and 3.4% after AgNO_3_ and p‐BQ were introduced, which indicated that e^−^ and $O_{2}^{-}$ played a primary role in the reaction process. While $O_{2}^{-}$ was generated through one‐step reduction of O_2_ by photoelectrons, and was the crucial intermediate of the H_2_O_2_ generation. Nevertheless, the elimination rate was found to decrease to 62.6% in the condition of bubbling Ar prior to illumination, thereby indicating that O_2_ played a significant role in the reaction (Figure 5a). The performance did not decline significantly, which can be partly attributed to residual oxygen in the system and partly to direct electron reduction, which operated independently of free radicals. The removal rates decreased to varying degrees under both UV and visible light, which were 82.5% and 43.3%, respectively. It was evident that the catalytic activity exhibited a more pronounced reduction under conditions of visible light (Figure 5b). This was attributable to the fact that CW‐2 primarily demonstrated strong absorption in the UV wavelength range according to UV–vis DRS results. Concurrently, the catalytic activity diminished significantly in the absence of MeOH with the removal rate of 8.4%, a phenomenon attributed to the substantial number of unconsumed holes resulting from recombination with photoelectrons, consequently reducing the charge utilization efficiency.

To determine the origin of the enhanced $O_{2}^{-}$ selectivity, we calculated the O_2_ adsorption energies for g‐C_3_N_4_ and CW‐2. While pristine g‐C_3_N_4_ showed weak adsorption (1.1 eV), the introduced Lewis‐acidic W^6+^ sites in CW‐2 strongly bonded to O_2_ with an energy of −2.38 eV. The electron‐withdrawing nature of these W sites thereby made O_2_ adsorption on the adjacent g‐C_3_N_4_ framework thermodynamically less favorable, raising its energy to 1.59 eV and confirming the W sites as the exclusive active centers for O_2_ activation (Figure 5c). Charge density difference of CW‐2 after O_2_ adsorption was investigated to explore the real reaction sites. As illustrated in Figure 5f, significant charge interactions were observed to occur between W and O_2_, rather than at the N or C sites, where charge accumulation occurred on W sites and depletion occurred on O sites. It had been demonstrated that the charge transfer from O_2_ 2p π^*^ antibonding to W 5d orbital enhanced the adsorption, thereby facilitating the activation of O_2_. This finding suggested that Lewis‐acidic W^6+^ sites function as electron acceptors and catalytic active sites, while g‐C_3_N_4_ served as the electron supplier. Such a functional segregation strategy, where different sites played distinct but complementary roles, has also been shown to accelerate multistep catalytic processes [53]. Electron paramagnetic resonance (EPR) tests were investigated using DMPO as a spin‐trapping reagent to explore the concentration of $O_{2}^{-}$. As illustrated in Figure 5d, CW‐2 exhibited a higher signal strength of $O_{2}^{-}$, indicating the enhanced selectivity toward $O_{2}^{-}$. To quantitatively compare the generation rates of $O_{2}^{-}$, nitro blue tetrazolium (NBT) colorimetric method was employed. As demonstrated in Figure S17, within a time frame of 16 min, CW‐2 displayed a removal rate of 96.6% for NBT that was only 4.3% for bare g‐C_3_N_4_. Evidence suggested that the introduction of W sites promoted Pauling‐type adsorption of O_2_ in the sample, thereby facilitating the radical reduction pathway mediated by the ORR.

The utilization of in situ diffuse reflectance infrared Fourier transform spectroscopy (DRIFTS) is pivotal in facilitating the detailed probing of free radical reaction pathways throughout ORR. As shown in Figure 5e, the adsorption peak at 1716 cm^−1^ was indicative of an adsorbed water molecule, while the peak at 1455 cm^−1^ was assigned to O─O stretching vibrations of surface O_2_ molecules [54]. Three bands according to the orbital hybridization between O_2_ and C─N bond, in which 1017 cm^−1^ was attributed to the C─O─O binding, while 1529 and 1577 cm^−1^ were assigned to N─O bonding [55]. It was evident that, due to the comparatively substantial specific surface area of g‐C_3_N_4_, the Yeager‐type O_2_ adsorption pattern was also observed between C─N bonds. However, upon light excitation, the Z‐scheme charge transfer mode facilitated the reduction of O_2_ adsorbed at W sites in Pauling type. The absorption peaks of some key free radicals gradually began to show up with increasing irradiation time. Peak at 932 cm^−1^ was recognized as the O─O stretching of peroxide species [56] and peak at 1105 cm^−1^ was deemed as the adsorbed $O_{2}^{-}$ [54]. Moreover, the peak located at 1229 cm^−1^ was attributed to ^*^OOH, which was the key intermediate formed after the hydrogenation of $O_{2}^{-}$ [57]. While peaks at 817 and 1046 cm^−1^ were attributed to the adsorbed ─OH that were generated through the water oxidation process. Notably, the appearance of $O_{2}^{-}$ and ^*^OOH simultaneous suggesting the production of H_2_O_2_ complied with 2e^−^ stepwise path.

Peaks in the region of 1120–1200 cm^−1^ was indicative of the OOH bending of H_2_O_2_, and peaks at 1310 and 855 demonstrated the presence of adsorbed free H_2_O_2_. While peaks at 1634 and 1382 represented the stretching vibrations of O─H bending in H_2_O_2_, these peaks made good evidence of the generation of H_2_O_2_ through ORR [54, 56, 57]. The generation process of both $O_{2}^{-}$ and H_2_O_2_ was revealed by in situ DRIFTS, with both potentially contributing to uranium extraction. Combined with the above analysis, the ORR pathway is listed as follows:

(1) $$\mathrm{Catalyst}+O_{2}\to \;*O_{2}$$

(2)

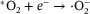

(3) $$\cdot O_{2}^{-}+H^{+}\to \cdot \mathrm{OOH}$$

(4) $$\cdot \mathrm{OOH}+H^{+}+e^{-}\to H_{2}O_{2}$$

As illustrated in Figure 5g, the Gibbs free energy of the usual U(VI) photocatalytic removal, including two‐electrons direct reduction of ${\mathrm{UO}}_{2}^{2+}$ to UO_2_, free radicals pathway (${\mathrm{UO}}_{2}^{2+}$ + 2 $O_{2}^{-}\;$→ UO_2_) as well as reacting with H_2_O_2_ to form uranium peroxide pathway (${\mathrm{UO}}_{2}^{2+}$ + H_2_O_2_ + *x*H_2_O → (UO_2_)O_2_·*x*H_2_O + 2H^+^) upon CW‐2 were calculated [1]. An energetically downhill tendency indicated each pathway was thermodynamically favorable. The pathway involving O_2_ activation to $O_{2}^{-}$ presented a lower energy barrier compared to the direct two‐electron reduction of ${\mathrm{UO}}_{2}^{2+}$, as evidenced by its more exothermic reaction energy. More energy requirements were observed for the further hydrogenation of $O_{2}^{-}$ compared to that of direct reaction with ${\mathrm{UO}}_{2}^{2+}$, indicating the H_2_O_2_ pathway may not be the efficient approach. In general, the free radical pathway demonstrated the least energy requirements, a feature that renders it more readily implementable. It was noteworthy that, in comparison with H_2_O_2_ generation through ORR, the free radical pathway involved a reduced number of reaction steps, thus indicating higher photoelectron utilization and diminished byproduct formation. And the efficient reduction reaction between $O_{2}^{-}$ and ${\mathrm{UO}}_{2}^{2+}$ occurred at the solid‐liquid interface, which suggested that the reduction products did not occupy active sites on the catalyst surface as observed in the direct two‐electrons reduction pathway, thereby circumventing any deleterious effects on catalytic efficiency. Consequently, sustained high catalytic activity was demonstrated.

XPS and XRD were employed to investigate the reduction productions. As illustrated in Figure S19, the U 4f XPS of the reduction products after four cycles revealed the presence of U(IV) species, characterized by 380.5 eV for U 4f_7/2_ and 391.4 eV for U 4f_5/2_. In addition, the presence of small amount U(VI) species was evident, as evidenced by the peaks at 381.8 and 392.5 eV, which corresponded to the U 4f_7/2_ and U 4f_5/2_ orbitals of U(VI), respectively [58, 59]. Quantitative analysis showed that U(IV) accounts for 92.2% of the total uranium products, indicating efficient reduction of U(VI). The signal of U(VI) species observed in XPS was attributed to the oxidation of UO_2_ to UO_2+x_ by ROS formed during ORR [5] or the conversion of ${\mathrm{UO}}_{2}^{2+}$ to uranium oxide hydrate ((UO_2_)O_2_·2H_2_O) in the function of H_2_O_2_ [39, 60]. This result suggested that, although the generation of H_2_O_2_ was thermodynamically unfavorable, trace amount of U(VI) species was still detected in the in situ DRIFTS results. As illustrated in Figure S20, the XRD patterns demonstrated that, following cycles, the crystal structure of CW‐2 was maintained, and there was an overlap with the diffraction peaks of U species. Peakes at 28.24°, 32.72°, 46.94°, and 55.70°, corresponding to the (111), (200), (220), and (311) facets of UO_2_ (#05‐0550), were consistent with XPS results [58]. The XPS and XRD results further confirmed the photoreduction of ${\mathrm{UO}}_{2}^{2+}$ to UO_2_ by CW‐2.

**FIGURE 6:**
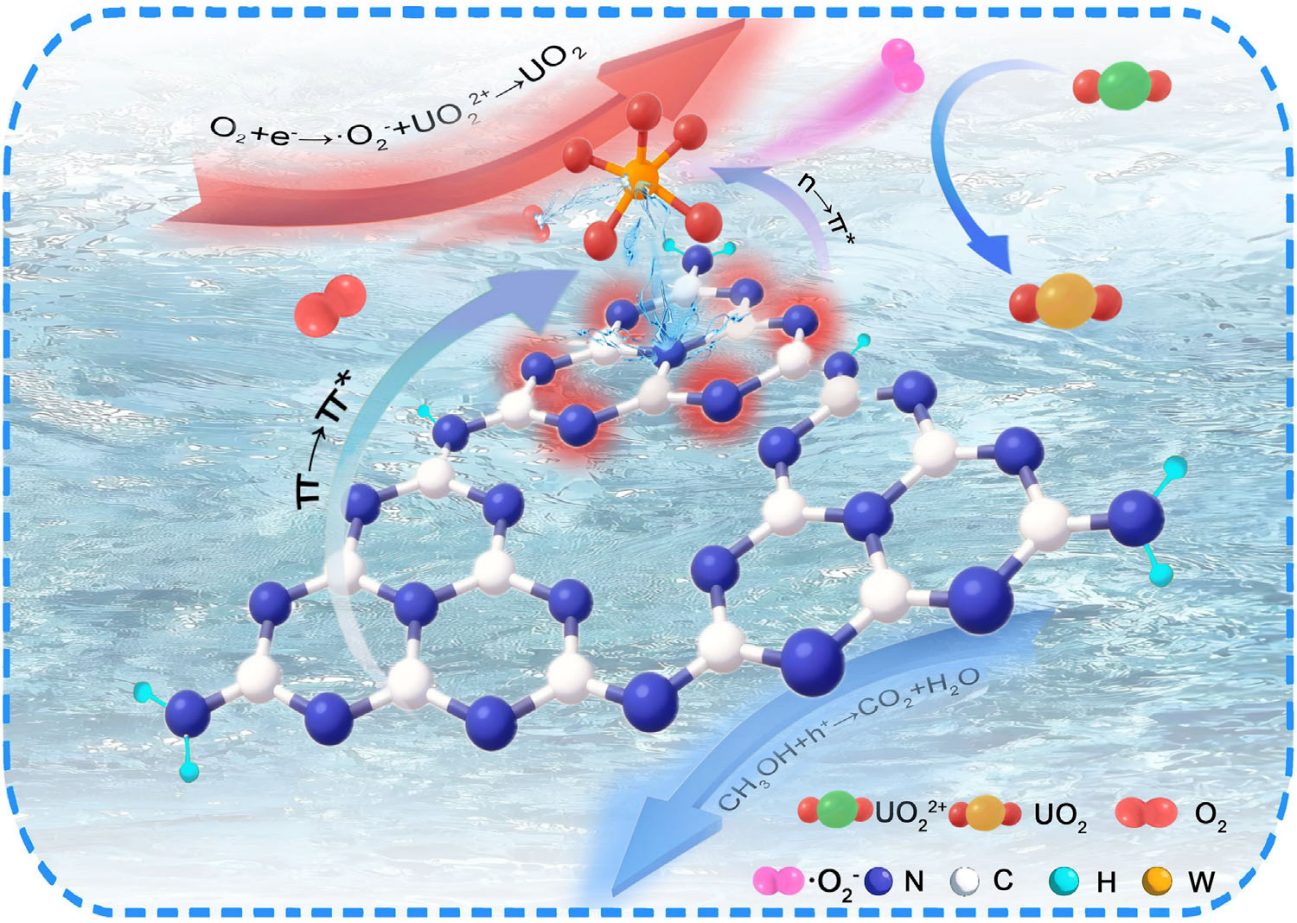
Mechanism of the radical‐mediated photocatalytic U(VI) reduction via W−N−C tandem centers on g‑C_3_N_4_.

In general terms, the enhancement of W−N 5d/2p covalence resulted in the distortion of the perfect tri‐s‐triazine structure and delocalization of electrons on g‐C_3_N_4_, thereby promoting the n → π^*^ and π → π^*^ transition. This, in turn, accelerated the excitation of photoinduced electrons. Moreover, the accumulation of electrons on N sites induced by higher electronegativity engendered an internal electric field and established Lewis‐acidic W^6+^ sites capable of accepting lone pair electrons from O 2p orbitals through Pauling‐type adsorption. Under light illumination, the internal electric field promoted the charge transfer from g‐C_3_N_4_ to WO_3‐x_ through W─N─C coordination, serving as the efficient charge transfer channel. Electrons generated by photoexcitation and enriched in W sites were observed to reduce adsorbed O_2_ to $O_{2}^{-}$. It had been ascribed to the reduced energy demands in comparison to those of the ensuing hydrogenation stage, which yielded H_2_O_2_. The $O_{2}^{-}$ had been observed to exert a direct influence on ${\mathrm{UO}}_{2}^{2+}$, leading to its reduction to UO_2_ (Figure 6).

## 3 Conclusion

In this study, a W‐N‐C tandem catalytic center was constructed by anchoring unsaturated WO_3‐x_ to the 3DOM g‐C_3_N_4_. This approach has been shown to achieve the enhancement of carrier separation, migration efficiency, and selectivity of the efficient radical pathway for U(VI) reduction. It has been demonstrated that charge enrichment on the incompletely oxidized W atoms migrated toward N due to its greater electronegativity, thereby promoting electron delocalization of g‐C_3_N_4_ and charge accumulation on N sites. The enhanced W/N 5d/2p hybridization caused charge redistribution and the distortion of the perfect tri‐s‐triazine structure. This, in turn, promoted the n → π^*^ and π → π^*^ transitions, thereby narrowing the bandgap and enhancing the utilization rate of visible light. More importantly, the formed Lewis‐acidic W site facilitated the O_2_ adsorption via a single line Pauling type, resulting in the enhancement of $O_{2}^{-}$ generation. The optimized sample CW‐2 demonstrated a photocatalytic removal efficiency of 98.6% for U within 16 min, which is 6.85 and 5 times higher than that of g‐C_3_N_4_ and WO_3‐x_, respectively. This strategy not only achieved minute‐scale uranium reduction via a radical‐mediated pathway but also eliminated the impractical requirement for oxygen exclusion. This strategy establishes a paradigm for designing high‐performance photocatalysts for uranium extraction.

## Funding

This research was supported by the Lingchuang Research Fund of China National Nuclear Corporation (CNNC‐LCKY‐2024‐083), the Fundamental Research Funds for the Central Universities (lzujbky‐2023‐stlt01), the National Natural Science Foundation of China (22276013), and the Beijing Municipal Natural Science Foundation (2242009).

## Conflicts of Interest

The authors declare no conflicts of interest.

## Supporting information




**Supporting file**: advs75637‐sup‐0001‐SuppMat.docx.

## Data Availability

The data that support the findings of this study are available on request from the corresponding author. The data are not publicly available due to privacy or ethical restrictions.
