# Supplementary material for: W‐N‐C Tandem Catalytic Centers Mediating Efficient Charge Transfer for the Enhanced Radical‐Path U(VI) Photoreduction
Source: Adv Sci (Weinh). 2026 May 8;13(44):e75637. doi: 10.1002/advs.75637 (PMC13336017; doi:10.1002/advs.75637)
Supplement: Supplementary file 1 [file ADVS-13-e75637-s001.docx]

Supporting Information

**W-N-C Tandem Catalytic Centers Mediating Efficient Charge Transfer for the Enhanced Radical-Path U(VI) Photoreduction**

*Zhiyao Wu^1a^, Jinhao Xu^2a^, Hao Fu^3^, Yuxiang Deng^4^, Nannan Wang^4^, Wenhua Zhang^5^, Peng Zhang^1^, Zhiwei Huang^1^, Zhenpeng Cui^1^, Shuxian Hu^2*^, Wangsuo Wu^1^, Duoqiang Pan^1*^*

**1. Experimental**

**1.1. Chemicals and materials**

Cyanamide (CH_2_N_2_, Macklin Biochemical Technology Co., Ltd.), Tetraethyl orthosilicate (TEOS) (C_8_H_20_O_4_Si, Macklin Biochemical Technology Co., Ltd.), Ethanol absolute (Macklin Biochemical Technology Co., Ltd.), Ammonium hydroxide solution (28-30%, Macklin Biochemical Technology Co., Ltd.), ammonium metatungstate ((NH_4_)_6_W_7_O_14_·5H_2_O, Macklin Biochemical Technology Co., Ltd.), Nafion perfluorinated resin solution (Suzhou Sinero Technology Co., Ltd.), Na_2_SO_4_ (Guangdong Guanghua Science and Technology Co., Ltd.) and HF (5% (v/v) in H_2_O, Macklin Biochemical Technology Co., Ltd.), p-benzoquinone (p-BQ) ( Macklin Biochemical Technology Co., Ltd.), AgNO_3_ (Macklin Biochemical Technology Co., Ltd.), EDTA-2Na (Macklin Biochemical Technology Co., Ltd.), tert-butyl alcohol (TBA) (Macklin Biochemical Technology Co., Ltd.), L-histidine (Macklin Biochemical Technology Co., Ltd.), nitro blue tetrazolium (NBT) (Macklin Biochemical Technology Co., Ltd.) all reagents are analytically pure. High-speed centrifuge (H1850, Xiangyi Centrifuge Instrument Co., Ltd., Changsha High-tech Industrial Development Zone), tube furnace (GSL-1750X-KS, Hefei Kejing Material Technology Co., Ltd.), muffle furnace (KSL-1700X-A5, Hefei Kejing Material Technology Co., Ltd.), ultrasonic cleaning machine (F-010S, Suzhou Maihong Electric Appliance Co., Ltd.), and multifunctional magnetic stirrer (ZNCL-DL, Shanghai Yuezhong Instrument Equipment Co., Ltd.) were used to synthesize the catalysts.
**1.2. Synthesis of catalysts**

*1.2.1. Synthesis of SiO_2_ nanosphere*

SiO_2_ template was synthesized through the widely reported ammonia method. Specifically, 5 ml of ammonia was added dropwise to a mixture of 90 ml anhydrous ethanol and 3 ml deionized water and stirred for 30 minutes to obtain a homogeneous solution. Subsequently, 2 ml of TEOS was added dropwise with stirring and the reaction continued for 12 h. As the reaction progressed, the solution gradually changed to white. The SiO_2_ sample was collected by centrifugation and then subjected to multiple washes with deionized water and anhydrous ethanol. Subsequent to these steps, it was dried within an oven set to 60 °C for a duration of 12 hours.

*1.2.2. Synthesis of 3DOM g-C_3_N_4_*

1g of SiO_2_ nanosphere were homogeneously dispersed in water by stirring. Subsequently, the homogeneously dispersed slurry was filtered and placed in an oven setting at 60 °C overnight to get the ordered SiO_2_ template. Then 1 g of cyanamide was dispersed in 0.5 ml of deionized water and sonicated for 10min. The obtained solution was added to the SiO_2_ template and placed in a vacuum oven set to 60 °C under vacuum conditions overnight with the objective of fully infiltrating the cyanamide into the template. Subsequently, the mixture was subjected to calcination in a tube furnace under Ar atmosphere at a heating rate of 2 °C/min at 550 °C for a duration of 3 hours. The solid product obtained was immersed in 100 mL of a 5% HF solution for a period of 12 hours, after which it was thoroughly washed with deionized water on multiple occasions. Thereafter, the product was placed in a vacuum oven set at 60 °C for a further 12 hours. Finally, the solid was calcined at 400 °C for 3 hours with the heating rate of 2 °C/min, which resulted in the formation of 3DOM g-C_3_N_4_.

*1.2.3. Synthesis of CW-x*

50 mg of 3DOM g-C_3_N_4_ was dispersed in 30 mL of deionized water, following which the mixture was sonicated for a period of 10 minutes. Subsequently, varying quantities of metatungstate were incorporated into the suspension and agitated for a period of 3 hours. In this experiment, CW-1, CW-2, and CW-3 represent the addition amount of metatungstate, which is 90, 130, and 170 mg, respectively. The precursors were collected by filtration and subsequently subjected to a vacuum oven at a temperature of 60 °C for a duration of 6 h. Finally, the obtained powder was annealed at 400 °C for 3 h with the heating rate of 2 °C/min in a muffle furnace to get CW-*x*.

*1.2.4. Synthesis of WO_3-x_*

WO_3-x_ was synthesized through a single-step thermal treatment involving the calcination of metatungstate in a muffle furnace at a temperature of 400 °C, with a heating rate of 2 °C/min for a duration of 3 hours.

**1.3 Characterizations**

The crystal information was detected by the X-ray diffraction (XRD, Rigaku D/MAX 2500 V, Rigaku Corporation, Japan) with the Cu Ka radiation (λ = 0.15418 nm, 40 kV and 100 mA). The microstructure and lattice arrangements were investigated by scanning electronic microscopy (SEM, Sigma 300, Carl Zeiss, Germany) and transmission electronic microscopy (TEM, JEM-2100F, JEOL, Japan). The elemental distribution of the samples was detected by using energy dispersive spectroscopy (EDS) of Oxford Instrument. The elemental microanalysis and atom binding states were examined by X-ray photoelectron spectroscope (XPS, ESCALAB 250XI, Waltham, USA) with an Al Kα radiator. The photoelectric performance of as prepared samples was estimated using an electrochemical workstation (CHI660D, Chenhua Instrument, China) with a three-electrode system. Pt and Ag/AgCl electrodes were selected as the counter and reference electrodes, while 7 mg of catalyst coated on pure uorine-doped tin oxide (FTO) glasses (1 × 2 cm^2^) acted as working electrodes, with 0.5 M Na_2_SO_4_ as the electrolyte solution. Transient photocurrent responses spectroscopy, EIS and Mott-Schottky curves were tested under the open circuit voltage and Mott-Schottky curves were tested using 500, 1000 and 1500 Hz to reduce errors. A full spectrum Xe lamp (PLS-SXE300+/UV, Beijing Perfectlight, 320 nm < λ < 780 nm) was adopted as the light source deposited 10 cm away from the testing system to realize the transient photocurrent responses spectroscopy experiment. UV-vis diffuse reflectance spectra (DRS, UV-3600Plus, SHIMADZU, Japan) were applied to investigate the light-harvesting ability of the catalysts. Photoluminescence spectra (PL, FL3C-111 TCSPC, HORIBA, Japan) was obtained to study the recombination degree of photoinduced electrons and holes upon different samples. N_2_ adsorption-desorption isotherms (Autosorb iQ-C-MP, Quantachrome Instruments, USA) were measured to test investigate the BET surface areas.

**1.4 X-ray absorption fine structure and soft X-ray absorption spectroscopy**

Static X-ray absorption fine structure (XAFS) analyses of the W L_3_-edge were conducted utilizing a commercial Laboratory-Based XAFS spectrometer (RapidXAFS 2M, Anhui Absorption Spectroscopy Analysis Instrument Co., Ltd.). X-rays were generated via a Mo target X-ray source operated at 20 kV and 20 mA. A Si (660) spherically bent crystal analyzer (SBCA) with a radius of curvature of 500 mm served as the monochromator, thereby ensuring a diffraction geometry approaching a 90-degree backscatter angle at the absorption edge. After monochromatization, the X-rays pass through the sample and were collected using a high-energy-resolution silicon drift detector (SDD) to obtain the X-ray intensity. The XAFS data were acquired in transmission mode. During the XAFS measurements, the position of the absorption edge (E_0_) was calibrated using a standard W foil sample, and all data collection occurred within a single time period.

Soft X-ray absorption spectroscopy (sXAS) measurements of C K-edge and N K-edge were measured in NSRL beamlines MCD-A and MCD-B (Soochow Beamline for Energy Materials) of the National Synchrotron Radiation Laboratory in China using total electron yield (TEY) mode. The photon energy step of C K-edge and N K-edge sXAS spectra were set at 0.1 eV and the vacuum degree of the test chamber was 5×10^-8^ mbar.

**1.5 Photocatalytic performance**

Photocatalytic experiments were conducted within quartz tubes, with a volume of 40 mL. In summary, 12.5 mg of catalysts were dispersed in a mixed solution comprising 10 mL of UO_2_^2+^ solution (concentration: 1 × 10^-3^ mol/L), 13 mL of deionized water and 2 mL of methanol, the resulting solid-liquid ratio was 0.5. The pH of this solution was adjusted to 5.0 by NaOH (0.2 mol/L) solution. A 300 W Xenon lamp (PLS-SXE300+/UV, Beijing Perfectlight, 320 nm < λ < 780 nm) was employed as the light source. The photocatalytic process was conducted in the presence of oxygen at room temperature. The quartz tubes were subjected to a 30-minute stirring process in a dark environment to achieve adsorption equilibrium prior to illumination. During the light phase, at three-minute intervals, 1.4 mL of fluid was extracted and filtered through a 0.45 µm Nylon membrane to obtain the sample solution during photoreactions. Arsenazo-III method was used to estimate the residual concentrations of U(VI) in solutions, which was analyzed through UV–vis absorption spectra. The removal rate r (%) was determined through eq. (1), where C_0_ and C_t_ represent the U(VI) concentration in the absence of catalysts and after t min irradiation, respectively. The extraction amount Q_e_ (mg/L) was determined through eq. (2), where C_e_, V and m represent the U(VI) concentration at the end of photocatalytic reaction, volume of solution and quantity of catalyst respectively.

$\text{r = }\frac{C_{t}-C_{0}}{C_{0}} \times100\%$	(1)

$Q_{e}\text{ = }\frac{{(C}_{0}-C_{e}) \times V}{m} \times100\%$	(2)

The impact of pH, cation, anion and initial U(VI) concentration was investigated under identical conditions, with the exception of a modification to the initial pH level (from 3 to 7), the incorporation of interfering ions and initial U(VI) concentration (2 × 10^-5^, 1.2 × 10^-4^, 2 × 10^-4^, 4 × 10^-4^, 6 × 10^-4^, 8 × 10^-4^ mol/L). All the experiments were conducted thrice or more to ensure accuracy.

Turnover frequency (TOF) was calculated using the following formulas^[1]^:

$\text{TON = }\frac{\text{Moles of evoluted products}}{\text{Moles of active sites on photocatalyst}}$	(1)

$\text{TOF =}\text{ }\frac{\text{TON}}{\text{Reaction time (}\text{h}\text{)}}$	(2)

**1.6 *In-situ* DRIFTS measurements**

The *In-situ* diffuse reflectance infrared Fourier transform spectroscopy (DRIFTS) was used to analyze the intermediate species during O_2_ adsorption and photoreduction. Each sample was firstly purged with high purity Ar gas for 30 minutes at room temperature to collect the background, then high purity O_2_ with H_2_O and MeOH (8 VOL%) vapor was introduced through a bubbler and obtained by 64 cumulative scans at room temperature with a resolution of 8 cm^-1^ for each scan, and all spectral data were recorded. After 10 minutes O_2_ adsorption, 300 W Xenon lamp (PLS-FX300HU, Perfectlight, China, 320 nm < λ < 780 nm) was turned on until the finish of data collecting.

**1.7 Detecting of** **·O_2_^-^**

Nitro blue tetrazolium (NBT) was utilized as the detection agent for ·O_2_^-^.^[2]^ Briefly, 15mg catalyst was added to 30 mL of 0.1 mmol/L NBT solution. Following a 20-minute period of irradiation, the suspension was taken out at 2-minute intervals and filtered through a 0.45 µm nylon membrane to get the reaction solution. NBT can be reduced to insoluble blue formazan by ·O_2_^-^, through analyze the remaining NBT in the filtered liquids at the absorption wavelength at 259 nm, the yields of ·O_2_^-^ can be obtained according to eq. (3).

NBT+ ·O_2_^-^+ 2H^+^ → Formazan + O_2_	(3)

**1.8 DFT calculation details**

All calculations were performed using first-principles density functional theory (DFT) as implemented in the Vienna Ab initio Simulation Package (VASP).^[3]^ The exchange-correlation functional was treated within the generalized gradient approximation (GGA) using the Perdew-Burke-Ernzerhof (PBE) functional, and the ion-electron interactions were described using the projector-augmented wave (PAW) pseudopotential.^[4]^ A heterojunction model of WO_3-x_@g-C_3_N_4_ was constructed using a 3×2×1 supercell of WO_3-x_ (001) and a 2×2×1 supercell of g-C_3_N_4_ (001), with a 15 Å vacuum layer added along the z-direction to prevent periodic interactions. A plane-wave energy cutoff of 400 eV was employed, and convergence criteria were set to 10^-5^ eV for electronic energies and 0.02 eV/Å for atomic forces. For Brillouin zone integration, k-point meshes of 3×3×1, 2×3×1, and 1×1×1 were adopted for the isolated WO_3-x_ (001) surface, isolated g-C_3_N_4_ (001) surface, and the WO_3-x_@g-C_3_N_4_ heterojunction, respectively.

**2. Result and discussion**

**Figure S1.** XRD patterns.

**Figure S2.** FT-IR spectrum.

**Figure S3.** N_2_ adsorption-desorption isotherms.

**Figure S4.** SEM image of 3DOM g-C_3_N_4_.

**Figure S5.** TEM image of CW-2.

**Figure S6.** TEM and HRTEM images of WO_3-x_.

**Figure S7.** EDS mapping images of CW-2.

**Figure S8.** (a) XPS survey spectrum. High-resolution XPS spectra of (b) C 1s (c) O 1s.

**Figure S9.** DFT calculated structure model of (a) g-C_3_N_4_, (b) WO_3-x_, (c) CW-2. The silver, brown, red and grey balls represent N, C, O and W respectively.

**Figure S10.** DFT calculated VBM/CBM charge density distribution of (a) g-C_3_N_4_, (b) WO_3-x_.

**Figure S11.** DFT calculated work functions of (a) g-C_3_N_4_, (b) WO_3-x_.

**Figure S12.** Zeta potential of CW-2.

**Figure S13.** Recyclability of CW-2 for the photocatalytic U(VI) removal.

**Figure S14.** DFT calculated pDOS of (a) g-C_3_N_4_, (b) WO_3-x_.

**Figure S15.** DFT calculated O_2_ adsorption model on (a) g-C_3_N_4_, (b) g-C_3_N_4_ sites of CW and (c) WO_3-x_ sites of CW-2.

**Figure S16.** XPS spectra of (a) W 4f, (b) N 1s and (c) C 1s of CW-2 before and after stability test.

.

**Figure S17.** Photocatalytic NBT removal efficiency of g-C_3_N_4_ and CW-2.

**Figure S18.** The diagram illustration of the energy band structure, formation of

internal electric field and the Z-scheme charge transfer model of CW-2.

**Figure S19.** XPS spectrum of U 4f after photocatalytic reaction.

**Figure S20.** XRD patterns of CW-2 before and after photocatalytic reaction.

**Table S1.** Comparative list of photocatalytic performance of CW-2 with previously reported catalysts.

| Catalysts | U(Ⅵ) concentration (mg/L) | Time (mins) | Capacity  (mg/g) | pH | Sacrificial agents | Dosage (g/L) | | Conditions |
| --- | --- | --- | --- | --- | --- | --- | --- | --- |
| 4% Co_3_O_4_/P-PHI^[5]^ | 20 | 30 | 99.6 | 7 | MeOH | 0.2 | 300 W xenon lamp with a filter (λ > 420 nm)  Ambient condition | |
| OKDCN-2^[6]^ | 50 | 180 | 242.1 | 5 | \ | 0.2 | 300 W xenon lamp with a filter (λ > 420 nm)  Ambient condition | |
| ZFO-2^[7]^ | 50 | 40 | 237.5 | 5 | MeOH | 0.2 | 300 W xenon lamp with a filter (λ > 420 nm)  Excluding O_2_ | |
| SCN-C_3_N_4_^[8]^ | 50 | 120 | 191.8 | \ | \ | 0.25 | 300 W xenon lamp  Ambient condition | |
| S-g-C_3_N_4_^[9]^ | 30 | 120 | 57 | 7 | \ | 0.5 | 350 W xenon lamp with a filter (λ > 420 nm)  Excluding O_2_ | |
| WO_2.78_^[10]^ | 200 | 120 | 507.2 | 4.8 | TA | 0.25 | 350 W xenon lamp with a 1.5 G filter | |
| TT-Por COF-Ni^[11]^ | 200 | 300 | 166 | 9 | \ | 1 | 40 W LED lamp (λ = 460 nm) | |
| PTrSO-2^[12]^ | 50 | 120 | 99.5 | 4 | MeOH | 0.5 | 300 W xenon lamp with a filter (λ > 400 nm)  Ambient condition | |
| CNH^[13]^ | 50 | 60 | 498 | 5 | MeOH | 0.1 | 300 W xenon lamp  Ambient condition | |
| TFA-TAT-COF-Q^[14]^ | 50 | 135 | 391.6 | 5 | \ | 0.125 | 300 W xenon lamp  Ambient condition | |
| GCN-3CS^[15]^ | 50 | 120 | 47.5 | 4 | \ | 1 | 500 W xenon lamp with a filter (λ > 400 nm)  Ambient condition | |
| TP-DDB^[16]^ | 50 | 120 | 295.4 | 5 | \ | 0.33 | 300 W xenon lamp  Ambient condition | |
| **CW-2** | **1 × 10^-4^ M** | **16** | **557.56** | **5** | **MeOH** | **0.5** | **300 W xenon lamp**  **Ambient condition** | |

**References**

[1]	X.-H. Liu, W. Xie, Y. Mao, M. Ce, Q. You, H. Yan, X. Zhang, G. I. N. Waterhouse, H. Huang, *Appl. Catal. B Environ. Energy* **2026**, *385*, 126311.

[2]	H. Zhang, W. Wei, K. Chi, Y. Zheng, X. Y. Kong, L. Ye, Y. Zhao, K. A. I. Zhang, *ACS Catal.* **2024**, *14*, 17654-17663.

[3]	J. F. G. Kresse, *Phys. Rev. B* **1996**, *54*, 11169.

[4]	K. B. J. P. Perdew, *Phys. Rev. Lett.* **1996**, *77*, 3865.

[5]	L. Shi, Y. Wu, Z. Chang, P. Jiang, Y. Hua, Q. Shi, S. Hou, H. Wang, *Appl. Catal. B Environ. Energy* **2025**, *378*, 125618.

[6]	L. Chen, Y. Gao, J. Lian, L. Li, D. Ding, Z. Dai, *Sep. Purif. Technol.* **2023**, *307*, 122873.

[7]	P. Liang, L. Yuan, H. Deng, X. Wang, L. Wang, Z. Li, S. Luo, W. Shi, *Appl. Catal. B Environ.* **2020**, *267*, 118688.

[8]	P. Wang, E. Hu, L. Wu, J. He, Y. Wang, Z. Ou, F. Yang, T. Chen, W. Zhu, *Appl. Catal. B Environ. Energy* **2025**, *371*, 125266.

[9]	C. Lu, P. Zhang, S. Jiang, X. Wu, S. Song, M. Zhu, Z. Lou, Z. Li, F. Liu, Y. Liu, Y. Wang, Z. Le, *Appl. Catal. B Environ.* **2017**, *200*, 378-385.

[10]	J. Lei, H. Liu, C. Yuan, Q. Chen, J.-A. Liu, F. Wen, X. Jiang, W. Deng, X. Cui, T. Duan, W. Zhu, R. He, *Chem. Eng. J.* **2021**, *416*.

[11]	L. Chen, J. Hang, B. Chen, J. Kang, Z. Yan, Z. Wang, Y. Zhang, S. Chen, Y. Wang, Y. Jin, C. Xia, *Chem. Eng. J.* **2023**, *454*, 140378.

[12]	F. Yu, Z. Zhu, S. Wang, J. Wang, Z. Xu, F. Song, Z. Dong, Z. Zhang, *Appl. Catal. B Environ.* **2022**, *301*, 120819.

[13]	Z. Liu, S. Yao, A. Zhang, Y. Li, Y. Fu, Q. Zhou, *Appl. Catal. B Environ.* **2023**, *338*, 123023.

[14]	C. Liu, Y. Wang, Z. Dong, Z. Zhang, X. Cao, Y. Zhai, Y. Liu, *Chem. Eng. J.* **2025**, *514*, 163078.

[15]	Y. Cai, M. Fang, Y. Xu, M. Wakeel, B. Hu, X. Wang, *Chem. Eng. J.* **2025**, *518*, 164670.

[16]	Y. Chen, S. Dong, Y. Zhao, Y. Wang, L. Liu, H. Zhang, J. Wang, X. Peng, *Sep. Purif. Technol.* **2026**, *380*, 135166.
